# Fecal Microbiota Transplantation From Healthy Donors Reduces Glycemic Variability in Streptozotocin‐Induced Diabetic Rats via Enhanced Hepatic Glycogen Synthesis

**DOI:** 10.1155/ije/8852077

**Published:** 2026-05-23

**Authors:** Min Dou, Jingwen Xu, Xinhua Ye, Juan Liu, Li Shi

**Affiliations:** ^1^ Department of Endocrinology, The Second People’s Hospital of Changzhou, The Third Affiliated Hospital of Nanjing Medical University, Changzhou, 213164, Jiangsu, China; ^2^ Graduate School of Bengbu Medical University, Bengbu, 233000, Anhui, China, hznu.edu.cn; ^3^ Department of Geriatrics, Renhe Hospital Affiliated With Shanghai University, Shanghai, 201999, China; ^4^ Department of Nutrition, The Second People’s Hospital of Changzhou, The Third Affiliated Hospital of Nanjing Medical University, Changzhou, 213164, Jiangsu, China

**Keywords:** blood glucose fluctuation, fecal microbiota transplantation, liver glycogen, short-chain fatty acids, Type 1 diabetes mellitus

## Abstract

**Background:**

Blood glucose fluctuations in patients with brittle diabetes have been a problem for clinicians. A recent study found that transplanting the intestinal flora of healthy people to patients with brittle diabetes can improve their blood glucose fluctuations. However, the underlying mechanism remains unclear.

**Methods:**

Streptozotocin‐induced diabetic rats were assigned to receive fecal microbiota transplantation (FMT) from healthy donors or to remain untreated, while normal rats received FMT from brittle diabetes donors or remained untreated. Groups included the normal control group (NC group), the diabetic group (DM group), normal rats with FMT from brittle diabetic patients (NC‐DMFMT group), and diabetic rats with FMT from normal individuals (DM‐NCFMT group). Blood glucose variability, rat liver glucokinase, and glycogen levels, as well as intestinal short‐chain fatty acid content, were detected in each group of rats. Gut microbiota composition was analyzed using 16S rDNA sequencing.

**Results:**

Compared with the standard deviation of blood glucose (SDBG) (1.664 ± 0.427 mmol/L) in the NC group, that of the DM group (6.879 ± 1.475 mmol/L) was higher. However, the DM‐NCFMT group reduced SDBG (4.387 ± 0.619 mmol/L) vs. the DM group (*p* < 0.05). Hepatic glycogen (27.57 ± 5.254 mg/L) was lower in the DM group than in the NC group (55.48 ± 9.467 mg/L) but increased in the DM‐NCFMT group (37.59 ± 1.283 mg/L) vs. the DM group. The abundance of Bifidobacteria in the DM group was decreased compared to that in the NC group. In contrast, *Bifidobacterium* abundance in the DM‐NCFMT group increased after standard human flora transplants (*p* < 0.05). Correlation and stepwise regression analysis indicated that Bifidobacteria reduced SDBG partly by promoting hepatic glycogen synthesis, with an effect share of 23.01%.

**Conclusion:**

Normal individual fecal microbiota transplantation improves glucose variability in DM rats, potentially mediated by enhanced hepatic glycogen synthesis.

## 1. Introduction

Blood glucose fluctuations indicate glucose status and have become an independent risk factor for diabetes and its complications [1]. Normal individuals maintain narrow glycemic variability, generally characterized by a glycemic variability of 2–3 mmol/L throughout the day. People with diabetes show greater fluctuations in blood glucose, especially brittle diabetes patients who are mainly Type 1 diabetes patients. Due to the severe reduction of the *β*‐cell function and the deficiency of autonomous regulation of glycemia, brittle diabetes patients have more significant day–night glucose fluctuations compared to Type 2 diabetes, making it difficult to control. Current clinical approaches for brittle diabetes struggle to control blood glucose fluctuations effectively. Therefore, new clinical treatments are necessary to reduce blood glucose fluctuations.

It has been found that gut flora composition differs between healthy individuals and patients with Type 1 diabetes mellitus (T1DM) [2]. T1DM patients exhibit reduced gut microbiota diversity, decreased beneficial bacteria, and increased pathogenic bacteria compared to healthy individuals, suggesting that disturbance of gut microbiota correlates with T1DM. A Chinese clinical study transplanted washed intestinal flora from healthy individuals into patients, confirming that gut flora transplantation improves blood glucose fluctuations in patients with brittle diabetes [3]. However, the potential mechanisms by which gut flora transplantation improves glucose fluctuations in brittle diabetes are unclear. Glycogen decomposition and synthesis are essential sources and destinations of blood glucose. A decrease in glycogen synthesis or an increase in glycogen decomposition can cause postprandial blood glucose increases and fasting hypoglycemia, leading to higher glucose fluctuations.

Therefore, to explore how intestinal flora regulates glycemic homeostasis in the body, this study observed variations in blood glucose fluctuation levels and function of organs involved in blood glucose regulation in vivo by transplanting normal human intestinal flora into T1DM rats and transplanting brittle diabetes patients’ intestinal flora into normal rats.

## 2. Materials and Methods

### 2.1. Animals

Forty‐four five week old male Sprague Dawley (SD) rats (Department of Production, Animal Centre of Nanjing Medical University) were fed in a specific pathogen‐free environment with light and dark cycles of 12/12 h. Temperature (20°C–25°C) and humidity (50%–70%) were maintained. All rats were allowed to eat and drink freely.

**FIGURE 1:**
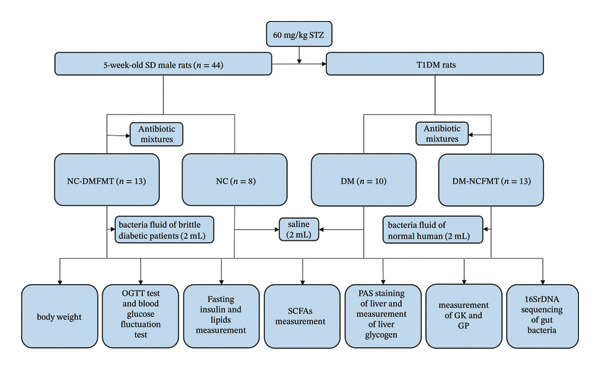
Experimental flow diagram. (NC, normal control group; DM, Type 1 diabetes group; NC‐DMFMT, normal rat transplanted with brittle diabetic flora group; DM‐NCMFT, T1DM rat transplanted with normal human flora group.) All experiments and protocols complied with the regulations of the institutional animal care and use committee of Nanjing Medical University (IACUC‐2310079).

One week after acclimatization, T1DM rats were induced by a single intraperitoneal injection of 60 mg/kg of streptozotocin (STZ) after 12 h of fasting. The control group rats were injected with an equivalent citrate buffer. 72 h after STZ injection, three nonconsecutive tail vein blood glucose measurements ≥ 16.7 mmol/L confirmed successful modeling. All 23 rats were successfully modeled. The experimental flow diagram is shown in Figure 1.

### 2.2. Gut Bacteria Liquid Preparation

Feces were collected from healthy individuals and patients with brittle diabetes who were hospitalized at the Department of Endocrinology of Changzhou Second People’s Hospital. Blood glucose levels of brittle diabetic patients, characterized by large fluctuations and fulfilling the criteria for diagnosing brittle diabetes [4], were collected from a Continuous Glucose Monitoring System (CGMS). The intestinal microbial suspension was extracted after an intelligent microbiological separation system (GenFMTer; FMT Medical Co. Ltd., Nanjing, China). Informed consent was obtained. The basic information of patients and normal individuals after admission is shown in Tables S1 and S2.

### 2.3. Oral Glucose Tolerance Test (OGTT) and Glucose Fluctuation Test

After 8 weeks of treatment, OGTT and glucose fluctuation tests were performed. Rats fasted for 12 h, gavaged with 50% glucose (2 g/kg) after baseline blood glucose measurement. Blood glucose levels in tail‐tip samples were measured using a blood glucose meter (AccuChek, Shanghai) at 30, 60, 90, and 120 min. The glucose tolerance was assessed by calculating the area under the glucose curve (AUC).

In the glucose fluctuation test [5], all rats were fasted overnight and administered a 50% glucose solution (2 g/kg) via gavage at 8:00, 12:00, and 16:00. Blood glucose levels were measured before each gavage, 30 min after each gavage, and throughout the night. The blood glucose was recorded at 8:00, 8:30, 12:00, 12:30, 16:00, 16:30, and 20:00. SDBG and Largest Amplitude of Glycemic Excursion (LAGE) were calculated from seven timepoints.

### 2.4. Methods

After blood collection, a 50% glucose solution was administered via gavage at a dose of 2 g/kg, and the liver specimens were removed 2 hours later. Enzyme‐linked immunosorbent assays were used to determine fasting insulin (FINS) levels in serum, as well as glucokinase (GK) and glycogen phosphorylase (GP) levels in the liver. Triglyceride (TG) and total cholesterol (TCH) levels in serum were measured using an automatic analyzer. Hepatic glycogen was assessed using the acid hydrolysis method, as described in the kit instructions, and periodic acid‐Schiff (PAS) staining, which was quantified using ImageJ.

The short‐chain fatty acids (SCFAs) from thawed colon contents were determined by gas chromatography. Sample separation was achieved on an Agilent DB‐FFAP capillary column (30 m × 250 × 0.25 μm) using a gas chromatograph. Mass spectrometry was performed on a 5977B MSD quadrupole mass spectrometer (Agilent Technologies). The mass spectrometer was operated under the following conditions: inlet temperature of 250°C, ion source temperature of 230°C, transfer line temperature of 250°C, and quadrupole temperature of 150°C. Electron ionization (EI) was applied with an electron energy of 70 eV, using a combination of full‐scan (SCAN) and selected ion monitoring (SIM) modes for analyte detection.

For quality assurance purposes, an equal‐volume aliquot of all samples was pooled to generate quality control (QC) samples. QC samples were injected at regular intervals throughout the sample batch to verify data stability and analytical reproducibility. Data acquisition and processing were carried out using the Agilent MSD ChemStation software, which was used for peak area extraction, retention time alignment, calibration curve plotting, and final quantitation of SCFA concentrations.

Rat fecal samples were tested for intestinal flora by 16S rDNA sequencing. DNA extraction was performed using the Magnetic Soil and Stool DNA Kit (TIANGEN, Beijing, China) according to the manufacturer’s instructions. Sequencing libraries were generated using the NEBNext Ultra DNA Library Prep Kit for Illumina (NEB, USA) following the manufacturer’s instructions, and index codes were added. Library quality was assessed using the Qubit 2.0 Fluorometer (Thermo Scientific) and the Agilent Bioanalyzer 2100 system. Finally, the library was sequenced on the Illumina NovaSeq 6000 platform, generating 250‐bp paired‐end reads. Sequence analysis was performed using the UPARSE software package with the UPARSE‐OTU and UPARSE‐OUT ref algorithms.

### 2.5. Statistical Analysis

Data were analyzed using IBM SPSS Statistics 27.0 and GraphPad Prism 8.0. Normally distributed data with homogeneous variance were subjected to one‐way analysis of variance. The Welch test was used for normally distributed data with heterogeneous variance, and the Kruskal–Wallis test was used for non‐normally distributed data. Stepwise regression and correlation analyses were used to identify correlations among different genera and serum indicators. Pearson’s correlation analyses were conducted on the normally distributed data, whereas Spearman’s correlation analyses were conducted on the non‐normally distributed data. *p* < 0.05 indicated statistical significance.

## 3. Results

### 3.1. Effect of FMT on Body Weight in Rats

Before the STZ injection, all rat groups had comparable body weights. Diabetic rats showed significant weight loss after STZ injection, while normal rats maintained steady growth. After standard colony transplantation (weeks 2–9), the DM‐NCFMT group rats exhibited slower weight decline than the DM group, while the NC‐DMFMT group had reduced weight gain compared to the NC group (Figure 2(a)). After the colony intervention for 8 weeks, the DM group weight (281.9 ± 22.17 g) was much lower than that in the NC group (536.1 ± 28.63 g) (*p* < 0.001), and the weight reduction of the DM‐NCFMT group (331.3 ± 48.87 g) was lower than that in the DM group (*p* < 0.05). In contrast, the NC‐DMFMT group (511.8 ± 34.41 g) lost less weight than the NC group; however, the difference was not statistically significant, as shown in Figure 2(b). These findings suggest that standard individual flora transplantation ameliorates weight loss in diabetic rats.

FIGURE 2Effect of FMT on the body weight of rats in all groups. (a) Trends in the body weight of rats in all groups. (b) Changes in the body weight at week 8 after FMT intervention (ns *p* > 0.05, ^∗^ *p* < 0.05, ^∗∗^ *p* < 0.01, ^∗∗∗^ *p* < 0.001).

**Figure:**
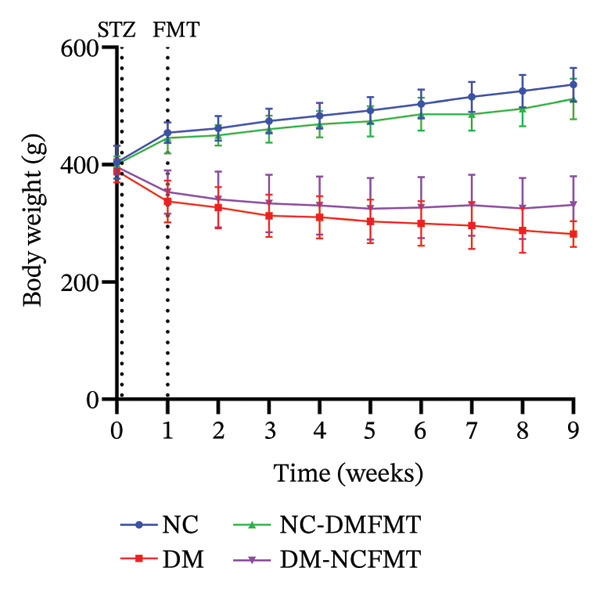
(a)

**Figure:**
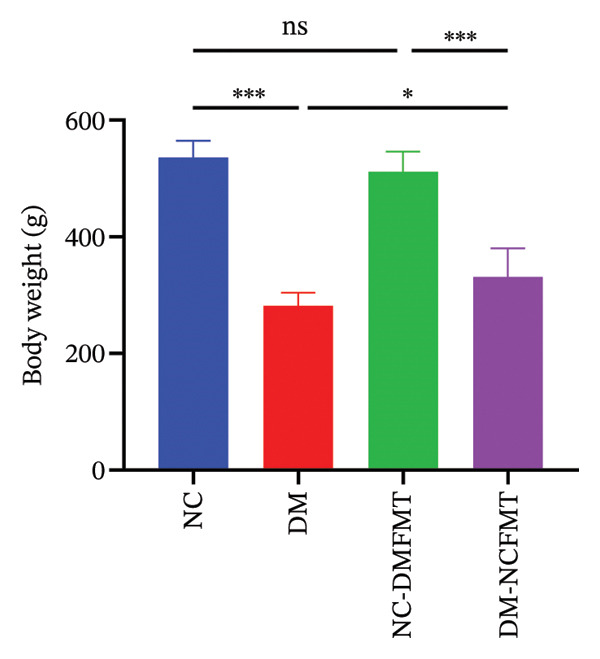
(b)

### 3.2. Effects of FMT on Glycolipid Metabolism in Rats

As indicated in Figures 3(a), 3(b), fasting blood glucose (FBG) was substantially higher in the DM group (26.10 ± 5.253 mmol/L) compared to that in the NC group (6.313 ± 0.514 mmol/L) and FINS (NC:14.43 ± 3.362 mmol/L, DM:7.984 ± 1.777 mmol/L) was lower, which were statistically different (*p* < 0.001), and after 8 weeks of FMT, FBG was slightly higher in the NC‐DMFMT group (6.455 ± 0.563 mmol/L) than in the NC group. There was a slight decrease in FINS levels compared to that in the NC group, but this difference did not reach statistical significance (*p* > 0.05). Compared to the DM group, FBG was reduced in the DM‐NCFMT group (21.10 ± 2.593 mmol/L) (*p* < 0.05), while FINS was not significantly different (*p* > 0.05). These findings suggest that insulin levels decreased in rats with T1DM, and normal individual FMT did not considerably improve insulin levels in T1DM rats.

FIGURE 3Effect of FMT on FBG (a), FINS (b), TG (c), and TCH (d) in rats of all groups.

**Figure:**
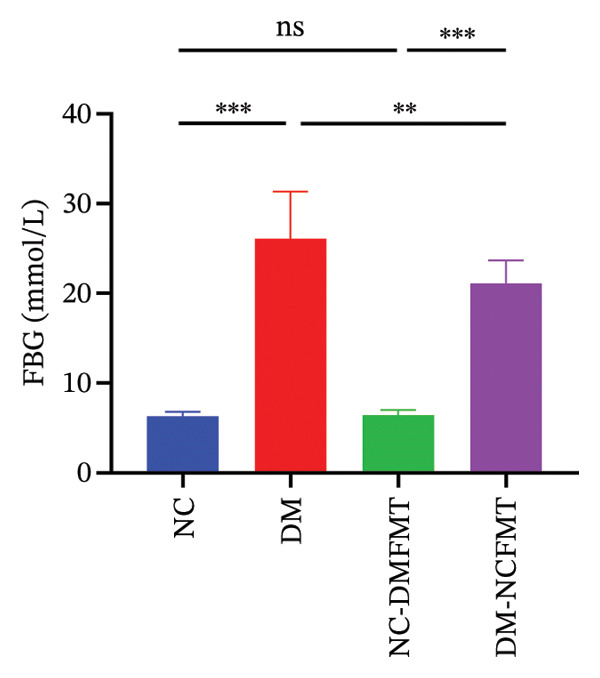
(a)

**Figure:**
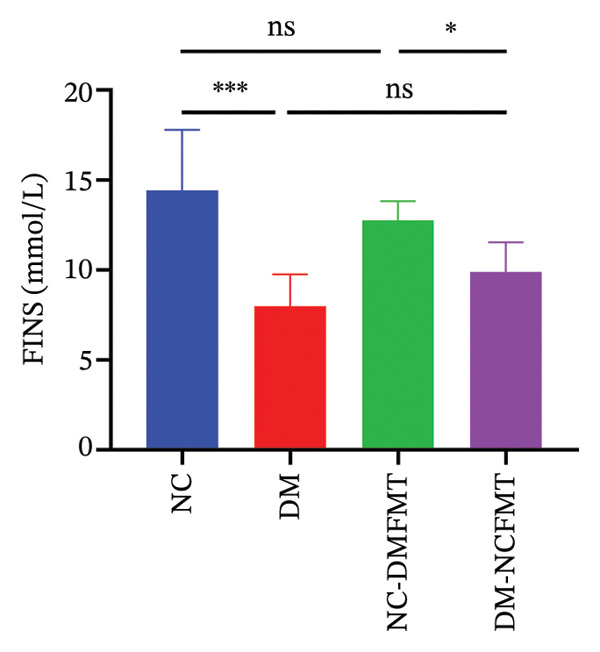
(b)

**Figure:**
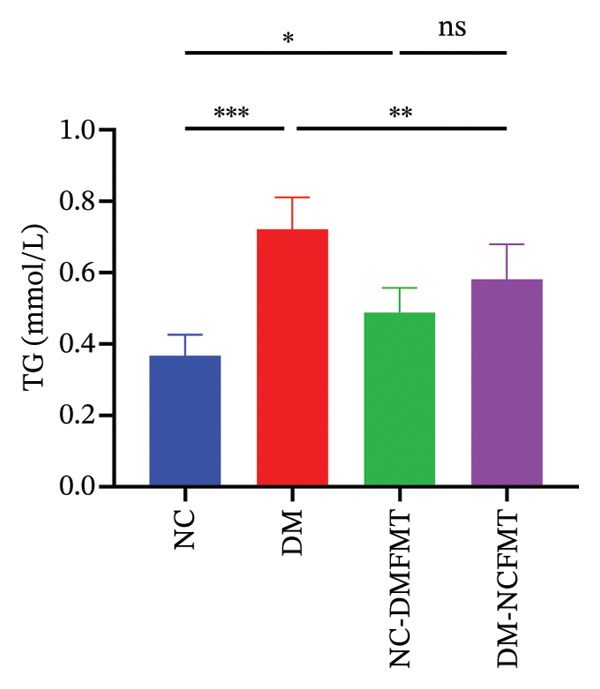
(c)

**Figure:**
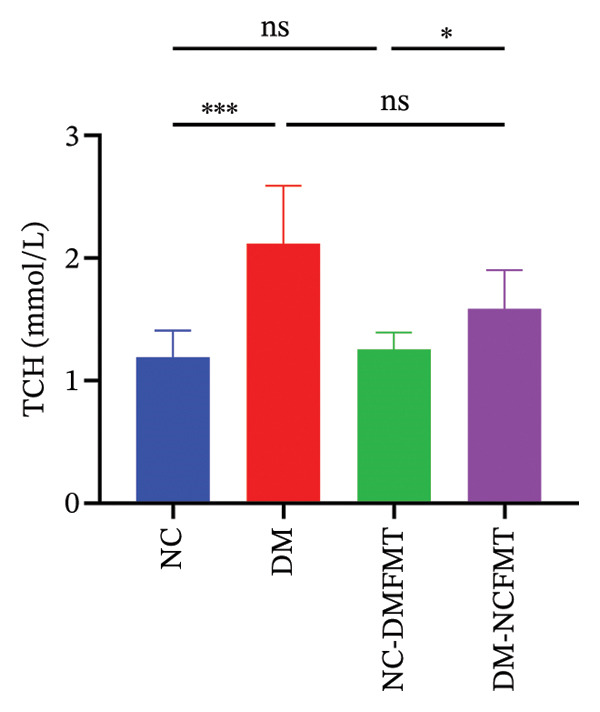
(d)

TCH and TG levels were markedly increased in the DM group (2.005 (1.765, 2.593) mmol/L, 0.722 ± 0.089 mmol/L) (Figures 3(c), 3(d)) than in the NC group (1.250 (0.940, 1.410) mmol/L, 0.367 ± 0.059 mmol/L) (*p* < 0.001). The TG level of the NC‐DMFMT group (0.489 ± 0.068 mmol/L) (*p* < 0.05) was markedly higher, and no statistical significance difference was observed in the TCH level (1.290 (1.143, 1.345) mmol/L) (*p* > 0.05) than in the NC group. Compared to the DM group, the DM‐NCFMT group (0.581 ± 0.099 mmol/L) showed significantly lower TG levels (*p* < 0.01); however, TCH was not significantly reduced (*p* > 0.05).

### 3.3. Effect of FMT on Glucose Tolerance in Rats

Figure 4 displays changes in blood glucose and AUC in the OGTT test. Before glucose gavage, the DM group (26.10 ± 5.253 mmol/L) had significantly higher blood glucose than the NC group (6.313 ± 0.514 mmol/L) (*p* < 0.01), and all groups exhibited initial increases followed by gradual declines after gavage of glucose. The DM group’s AUC_OGTT_ was dramatically higher (3751 ± 154.6 mmol/L∗min) than the NC group’s (1005 ± 129.7 mmol/L∗min) (*p* < 0.001). The DM‐NCFMT group demonstrated lower blood glucose than the DM group at 0 min (21.10 ± 2.593 vs. 26.10 ± 5.253) and 120 min (23.64 ± 4.904 vs. 31.33 ± 1953) (*p* < 0.01), and the AUC_OGTT_ was also lower (3158 ± 524.9 vs. 3751 ± 154.6) (*p* < 0.001). In contrast, the NC‐DMFMT group showed slightly higher blood glucose levels at 60–120 min and AUC_OGTT_ than the NC group, but these differences lacked statistical significance (*p* > 0.05). Meanwhile, we performed a two‐way analysis of variance on the AUC_OGTT_. We used AUC_OGTT_ as the dependent variable and then observed whether there were significant differences in the mean AUC_OGTT_ values for the two independent variables: diabetes and gut microbiota. The results showed that the F‐values for diabetes and gut microbiota were 644.68 and 10.69, respectively, with corresponding *p* values of *p* < 0.001 and 0.002, indicating statistically significant differences. Furthermore, the adjusted *R* ^2^ = 0.948, indicating that diabetes and gut microbiota can explain 94.8% of the variation in AUC_OGTT_ between groups. These findings suggest that transplantation of normal human flora improves glucose tolerance in diabetic rats.

FIGURE 4Effect of FMT on the OGTT of rats in all groups. (a) Oral glucose tolerance test. (b) Area under the glucose curve.

**Figure:**
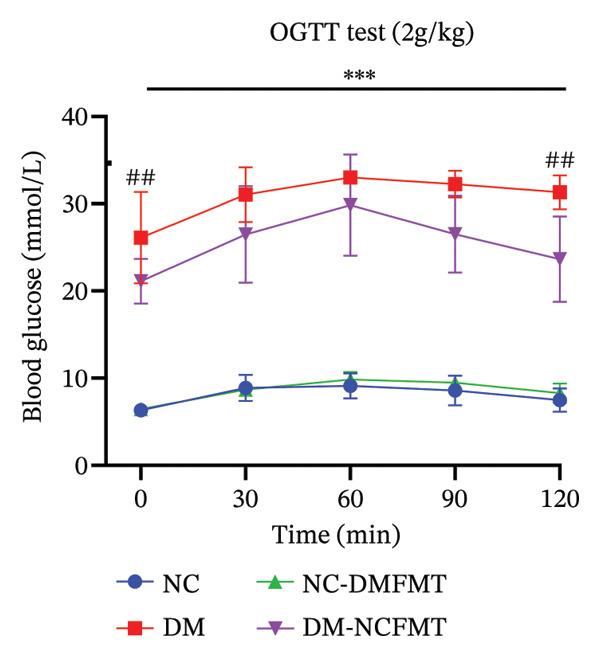
(a)

**Figure:**
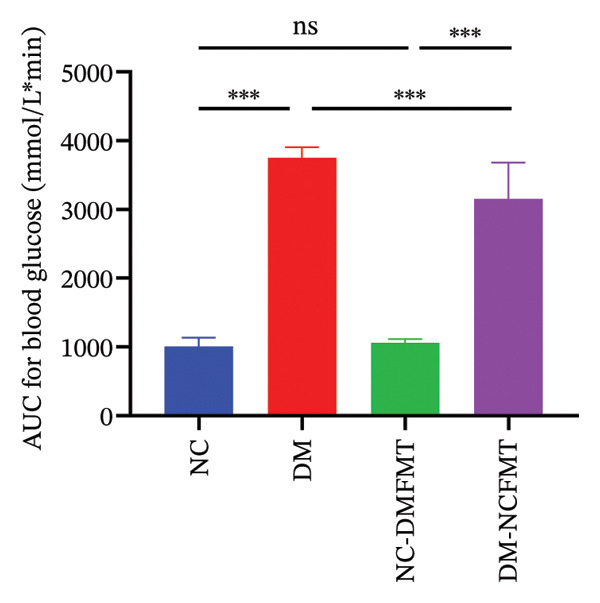
(b)

### 3.4. Effects of FMT on Blood Glucose Fluctuation in Rats

As Figure 5(a) and B shows, the DM group had higher SDBG and LAGE levels (6.879 ± 1.475, 16.78 ± 3.905 mmol/L) than the NC group (1.664 ± 0.427, 5.071 ± 1.389 mmol/L) (*p* < 0.001). SDBG in the NC‐DMFMT group (2.886 ± 0.551 mmol/L) (*p* < 0.05) had higher levels than in the NC group; however, LAGE levels showed no significant difference (*p* > 0.05). In contrast, the DM‐NCFMT group exhibited reduced SDBG and LAGE levels (4.387 ± 0.619 mmol/L, 11.83 ± 1.965 mmol/L) than the DM group (*p* < 0.05).

FIGURE 5Effect of FMT on the blood glucose fluctuation of rats in all groups. (a) The standard deviation of blood glucose (SDBG). (b) LAGE.

**Figure:**
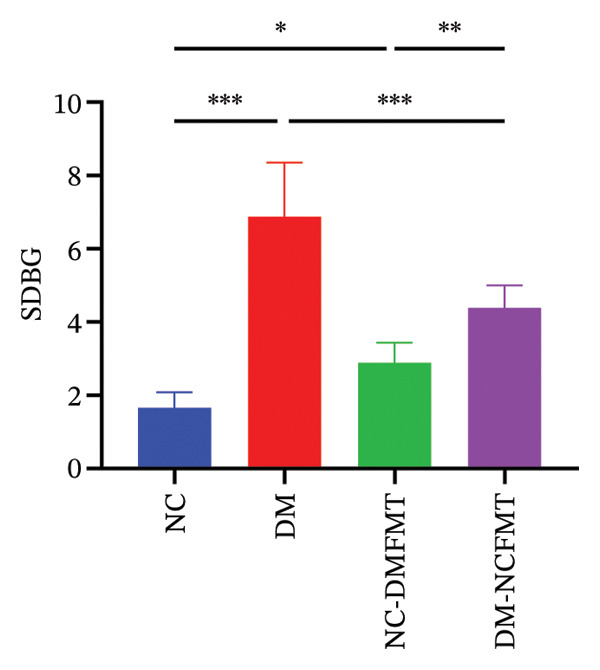
(a)

**Figure:**
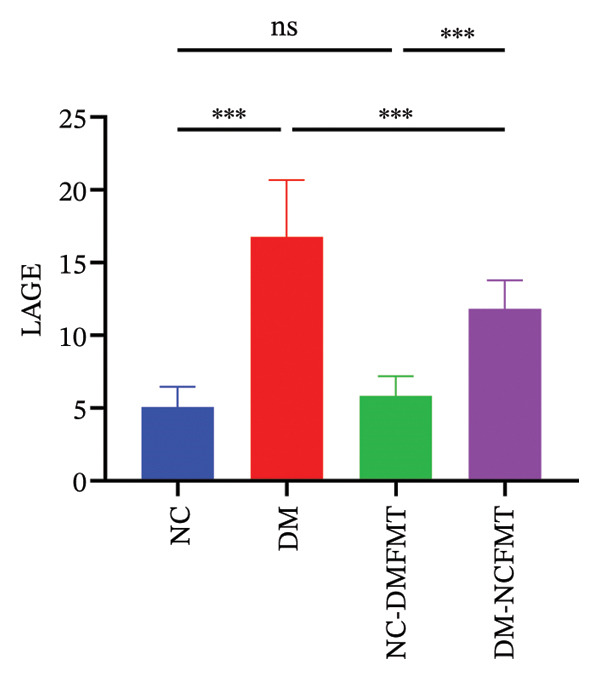
(b)

### 3.5. Effects of FMT on Hepatic Glycogen Synthesis in Rats

Liver glycogen in the NC group showed abundant purplish‐red particles with uniform distribution (Figure 6(a)), while DM rats exhibited paler, fewer, and uneven particles. The DM‐NCFMT group exhibited increased glycogen density and improved distribution compared to the DM group. Still, those in the NC‐DMFMT group were reduced and more disordered than those in the NC group. ImageJ quantification performed on liver glycogen staining sections revealed that the average gray value in the DM group (174.6 ± 7.880) was substantially lower than in the NC group (186.2 ± 3.840) (*p* < 0.001; Figure 6(b)). In contrast, the NC‐DMFMT group (180.5 ± 2.450) was decreased compared to the NC group (*p* < 0.05). However, this indicator was higher in the DM‐NCFMT group (180.8 ± 2.214) compared to that in the DM group (*p* < 0.05).

FIGURE 6Effect of FMT on liver glycogen of rats in all groups. (a) Liver glycogen PAS staining (40×). (b) Average optical density. (c) Liver glycogen level.

**Figure:**
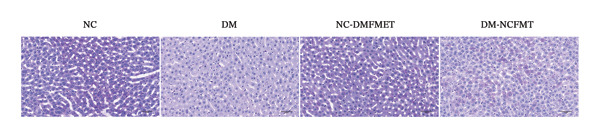
(a)

**Figure:**
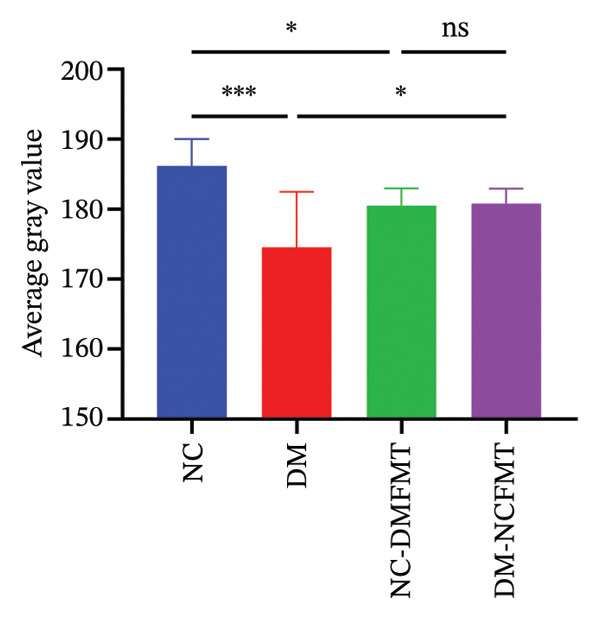
(b)

**Figure:**
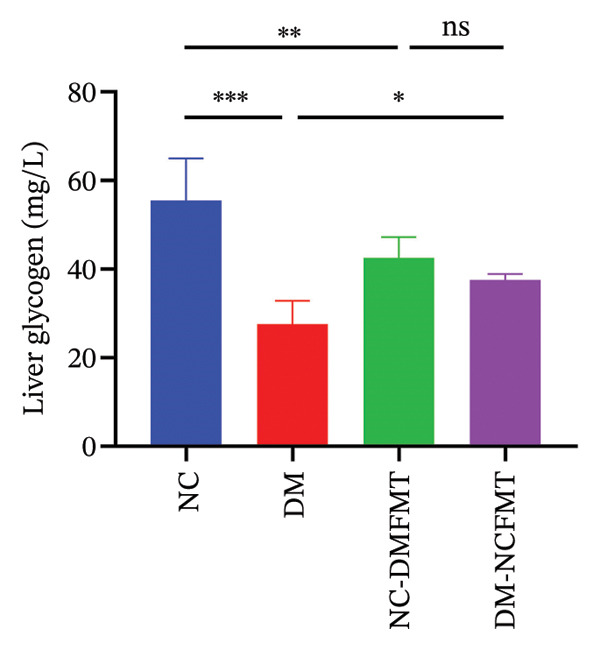
(c)

By measuring the hepatic glycogen content, the DM (27.57 ± 5.254 mg/L) and NC‐DMFMT (42.59 ± 4.665 mg/L) groups were lower than in the NC group (55.48 ± 9.467 mg/L) (*p* < 0.001, *p* < 0.01); the DM‐NCFMT group (37.59 ± 1.283 mg/L) showed higher hepatic glycogen content than the DM group (*p* < 0.05) (Figure 6(c)).

GK levels in the DM group (12.62 ± 1.367 pg/mL) were reduced with a significant difference from the NC group (17.60 ± 0.894 pg/mL) (*p* < 0.001). After FMT, the DM‐NCFMT group (14.97 ± 1.288 pg/mL) showed a marked increase in the GK level versus the DM group (*p* < 0.05). The GK level was lower in the NC‐DMFMT group (15.29 ± 0.998 pg/mL) compared with that in the NC group (*p* < 0.05; Figure 7(a)). As shown in Figure 7(b), liver GP levels were elevated in the DM (13.95 ± 1.104 pg/mL) and NC‐DMFMT (12.79 ± 1.186 pg/mL) groups compared to those in the NC group (12.14 ± 1.730 pg/mL), although the differences were nonsignificant (*p* > 0.05). The DM‐NCFMT group (13.40 ± 1.086 pg/mL) had slightly lower GP levels than the DM group and did not reach significance (*p* > 0.05).

FIGURE 7Effects of FMT on enzymes related to liver glucose metabolism in rats of all groups. (a) Glucokinase level; (b) glycogen phosphorylase level.

**Figure:**
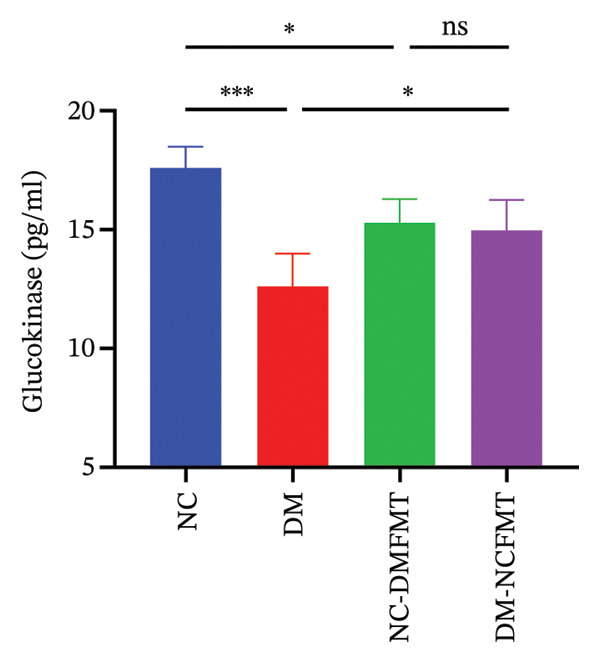
(a)

**Figure:**
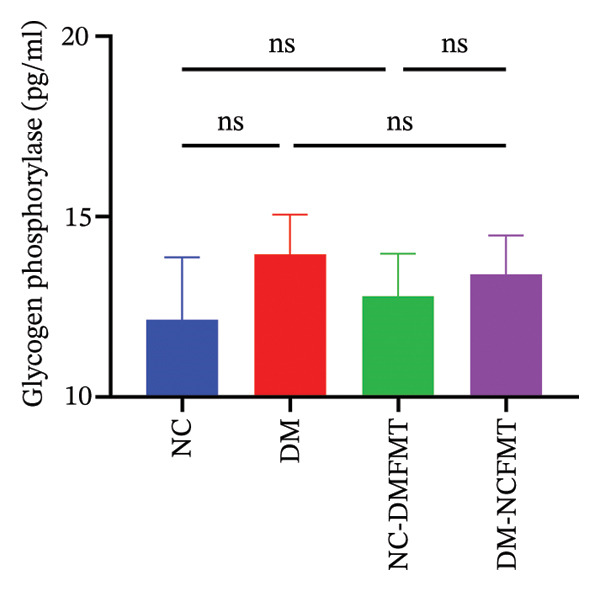
(b)

### 3.6. Effects of FMT on SCFAs in Rats

As Figure 8(a), 8(b), and 8(c) shows, the DM group (400.2 ± 126.8 μg/g, 177.3 (95.38,298.1) μg/g, 175.8103.3 μg/g) showed lower acetic acid, propionic acid, and butyric acid levels than the NC group (1004 ± 226 μg/g, 706.6 (492.5,1037) μg/g, 563.5 ± 91.69 μg/g) (*p* < 0.001), whereas valeric acid, hexanoic acid, isobutyric acid, and isovaleric acid failed to reach a statistical difference (*p* > 0.05; Figure S1). Acetic acid and butyric acid levels also exhibited a reduction in the NC‐DMFMT group (793 ± 96.32, 452.6 ± 37.79 μg/g) than in the NC group (*p* < 0.05). The levels of acetic acid and butyric acid were substantially higher in the DM‐NCFMT group (677.7 ± 187.1, 301.8 ± 78.47 μg/g) than in the DM group (*p* < 0.01, *p* < 0.05). However, other SCFAs show no statistically significant differences (*p* > 0.05; Figure S1). These findings suggest that FMT can modify intestinal SCFAs levels in diabetic and normal rats.

FIGURE 8Effect of FMT on short‐chain fatty acids in the colonic contents of rats in all groups. (a) Acetic acid. (b) Propionic acid. (c) Butyric acid.

**Figure:**
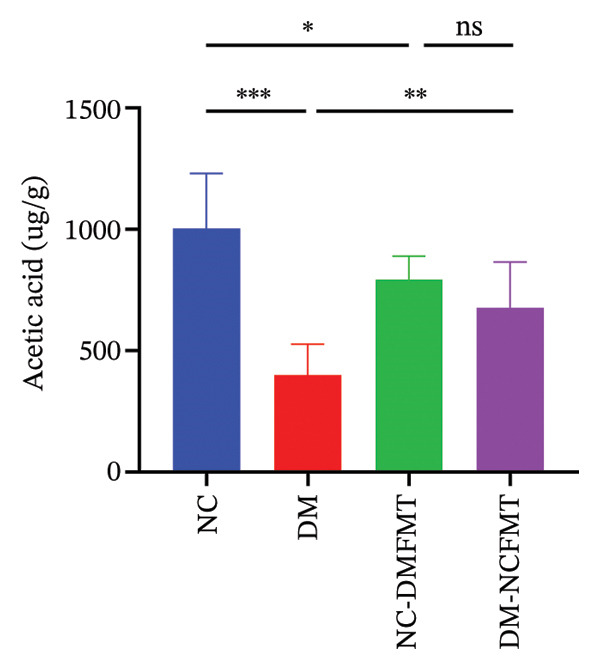
(a)

**Figure:**
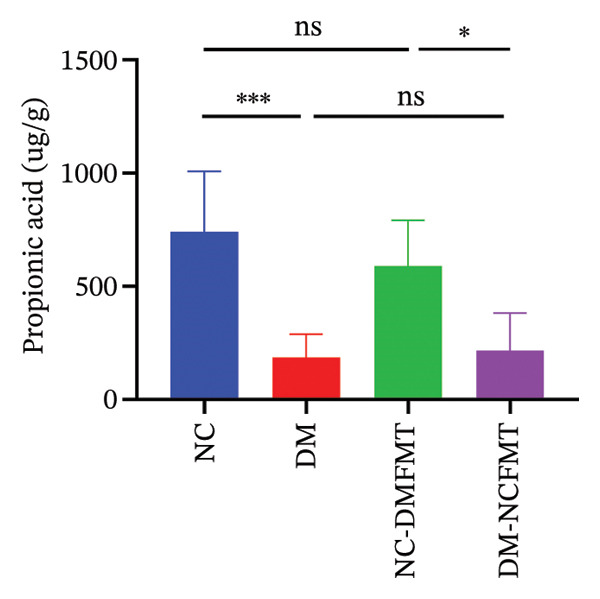
(b)

**Figure:**
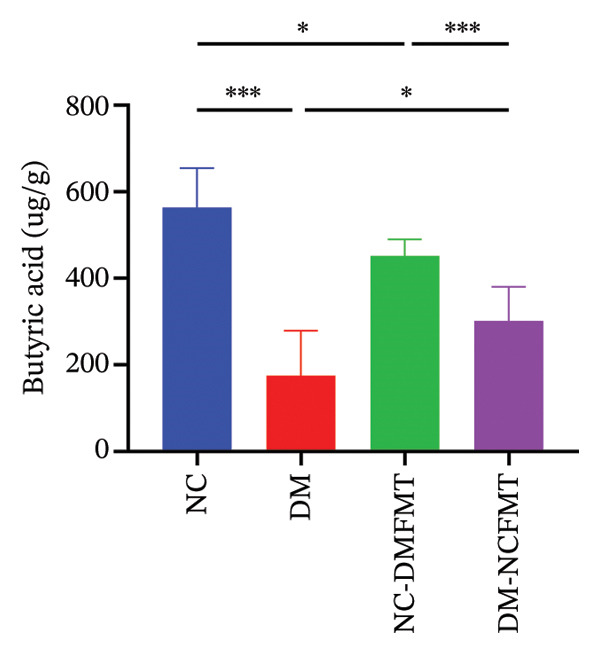
(c)

### 3.7. Effects of FMT on Gut Microbial Composition in Rats

To assess between‐sample community differences, we performed microbial beta diversity analyses by calculating weighted‐UniFrac distances. Based on Figure 9(a), the weighted‐UniFrac analysis revealed no significant differences between the NC and DM groups (*p* = 0.301). However, after FMT, the beta diversity significantly increased in the DM‐NCFMT group compared to that in the DM group (*p* = 0.0017) and in the NC‐DMFMT group compared to that in the NC group (*p* = 0.0081).

FIGURE 9Effects of FMT on the gut microbial composition in rats. (a) Weighted‐UniFrac beta‐diversity across groups. (b) Histogram of the TOP 10 species composition at the genus level of rats in all groups. (c) Histogram of LefSe analysis of differential species between DM and DM‐NCFMT groups. (LDA score more significant than 2 for differential flora).

**Figure:**
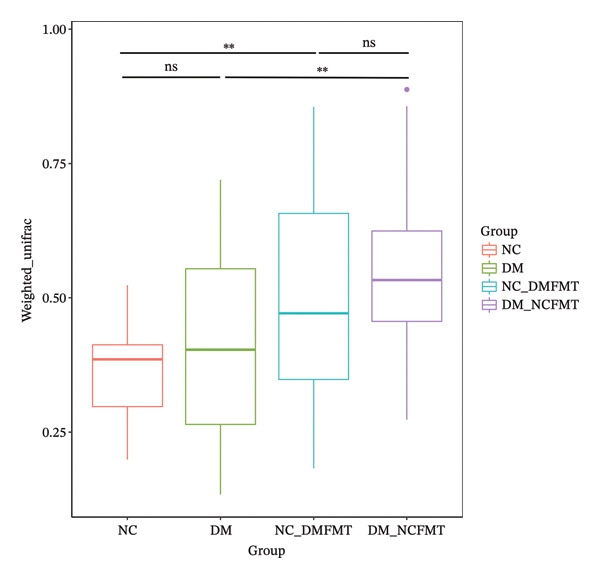
(a)

**Figure:**
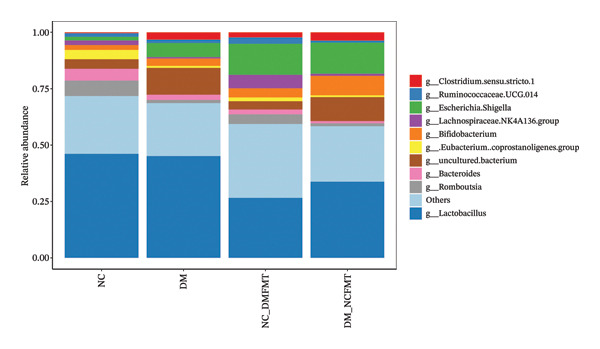
(b)

**Figure:**
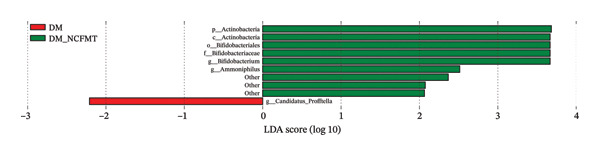
(c)

At the phylum level, Firmicutes, Bacteroidetes, Proteobacteria, and Actinobacteria dominated in all groups (NC group: 79.41%, 11.41%, 3.12%, 4.31%; DM group: 68.09%, 19.84%, 7.42%, 3.72%; NC‐DMFMT group: 67.49%, 8.04%, 16.79%, 5.62%; DM‐NCFMT group: 58.0%, 15.56%, 15.97%, 9.19%). The relative abundance of the four phyla in the DM and NC‐DMFMT groups was not significantly different than in the NC group (*p* > 0.05). The DM‐NCFMT group displayed increased Actinobacteria vs. the DM group (*p* < 0.05) (Figure S2).

At the genus level, Lactobacillus, Escherichia‐Shigella, uncultured bacterium, and *Bifidobacterium* were predominant (NC group: 46.10%, 1.63%, 4.21%, 2.17%; DM group: 45.13%, 6.21%, 11.82%, 3.30%; NC‐DMFMT group: 26.61%, 13.80%, 3.60%, 4.08%; DM‐NCFMT group: 33.76%, 13.80%, 10.60%, 8.61%) (Figure 9(b)). Compared with the NC group, the DM group showed increased *Clostridium sensu stricto* 1, Escherichia‐*Shigella*, and uncultured bacteria, and a reduction in Romboutsia (*p* < 0.05). The NC‐DMFMT group had a reduced abundance of Lactobacillus (*p* < 0.05), and the DM‐NCFMT group showed increased *Bifidobacterium* abundance (*p* < 0.05) versus the DM group.

LefSe analysis revealed higher *Ammoniphilus* and *Bifidobacterium* abundance (*p* < 0.05) but lower Candidatus_Profftella in the DM‐NCFMT group than in the DM group at the genus level (*p* < 0.05; Figure 9(c) and Figure S3). The LDA score of more than two is considered a differential colony.

### 3.8. Correlation Analysis

To investigate whether the improvement of abnormal blood glucose fluctuations in T1DM rats is related to SCFAs and hepatic glycogen, a correlation analysis was performed in four groups. The correlation analyses focused on acetic and butyric acids (group‐wise significant variables). As depicted in Figures 10(a), 10(b), the correlation coefficients between SDBG, LAGE, and acetic acid were −0.7571 and −0.7444 (all *p* < 0.0001). While the correlation coefficients between SDBG, LAGE, and butyric acid were −0.8563 and −0.8318 (all *p* < 0.0001; Figures 10(c), 10(d)), acetic acid, butyric acid, and SDBG, LAGE all showed a strong negative correlation. The correlation coefficients between SDBG, LAGE, and hepatic glycogen content were −0.8951 and −0.8826, with *p* values of < 0.0001, suggesting a negative correlation (e.g., Figures 10(e), 10(f)). In addition, according to Figures 10(g), 10(h), a positive correlation existed between acetic acid, butyric acid, and hepatic glycogen (*r* = 0.8518; *r* = 0.8043) (*p* < 0.0001). These findings suggest reduced glucose variability associated with elevated SCFAs and hepatic glycogen, indicating their synergistic roles in mitigating diabetic glycemic instability.

**FIGURE 11:**
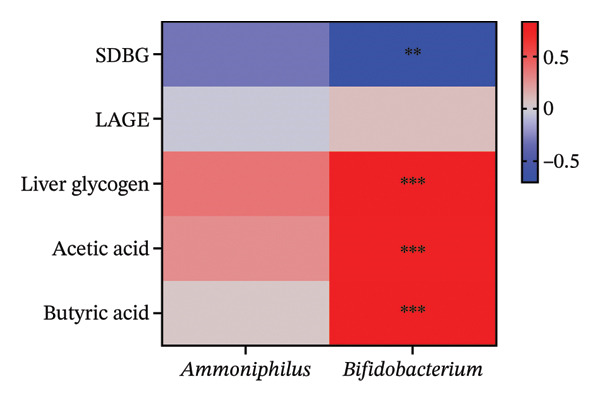
Correlation analysis of differential flora and clinical indicators between DM and DM‐NCFMT groups at the genus level.

FIGURE 10Correlation analysis. Based on 16SrDNA results, *bifidobacterium* and *ammoniphilus* at the genus level differed significantly between DM and DM‐NCFMT groups, so in this experiment, the two different genera of bacteria between DM and DM‐NCFMT groups were analyzed for correlation with the blood glucose fluctuation level, liver glycogen, acetic acid, and butyric acid (Figure 11). Correlation analysis revealed that *ammoniphilus* showed no associations with SDBG, LAGE, hepatic glycogen, acetic acid, and butyric acid (*r* = −0.3115, −0.04628, 0.3709, 0.2731, 0.04979; all *p* > 0.05). While *bifidobacterium* negatively correlated with SDBG (*r* = ‐ 0.7030, *p* = 0.0011), positively correlated with hepatic glycogen, acetic acid, and butyric acid (*r* = 0.8305, 0.7780, and 0.8196, respectively, *p* = 0.0008, 0.0001, and < 0.0001), and there was no correlation with LAGE (*r* = 0.08925, *p* = 0.7247).

**Figure:**
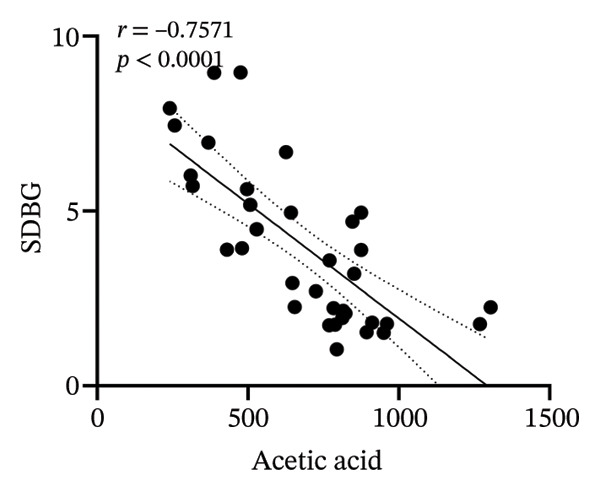
(a)

**Figure:**
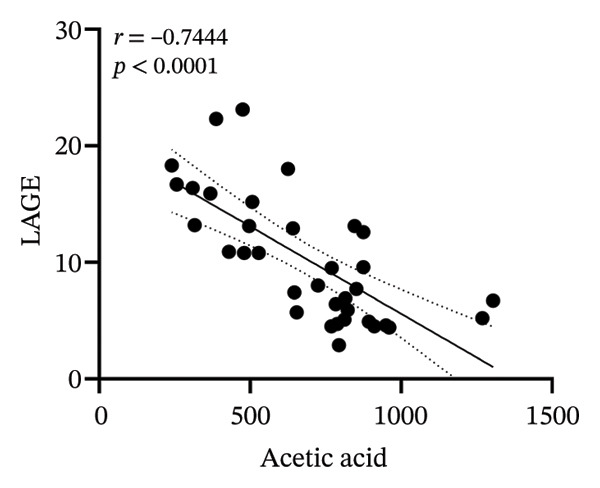
(b)

**Figure:**
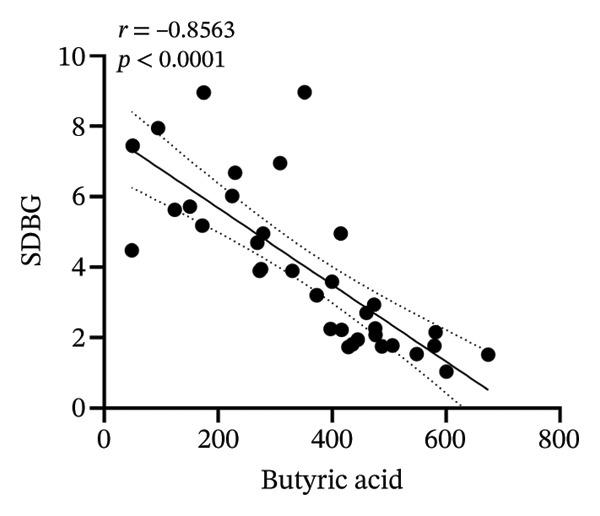
(c)

**Figure:**
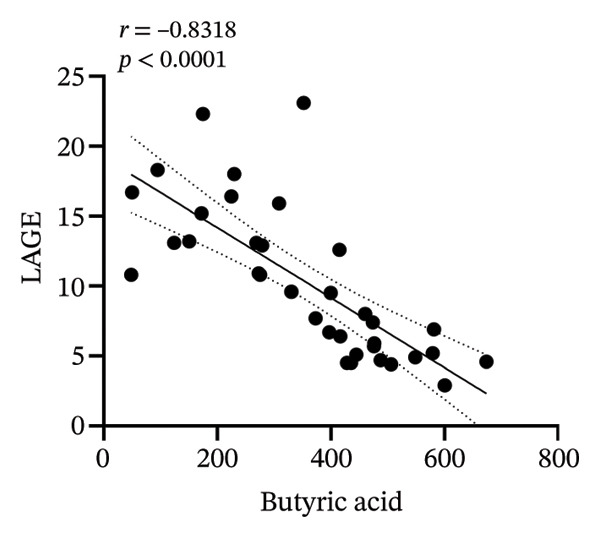
(d)

**Figure:**
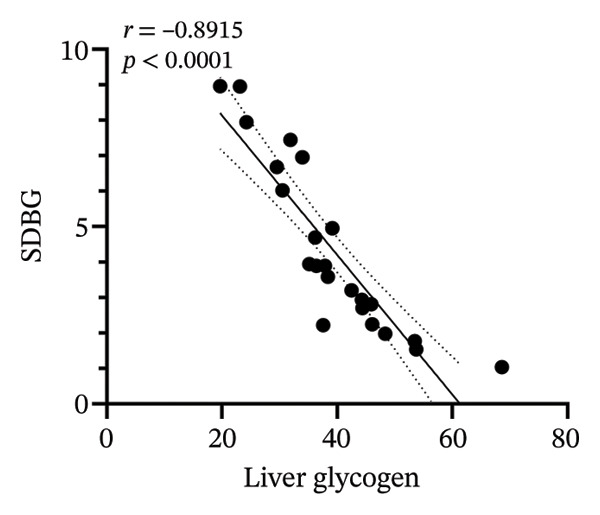
(e)

**Figure:**
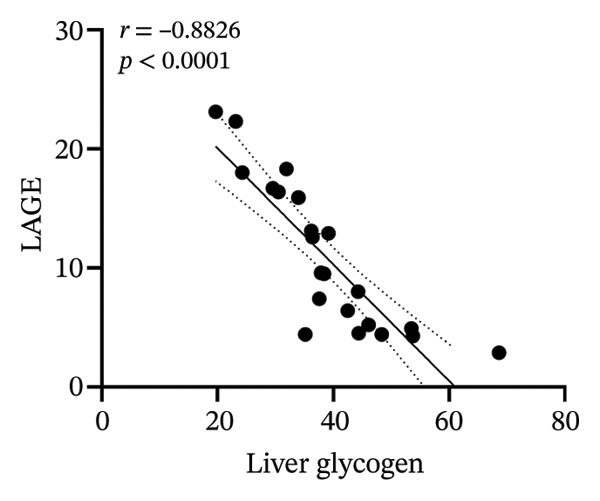
(f)

**Figure:**
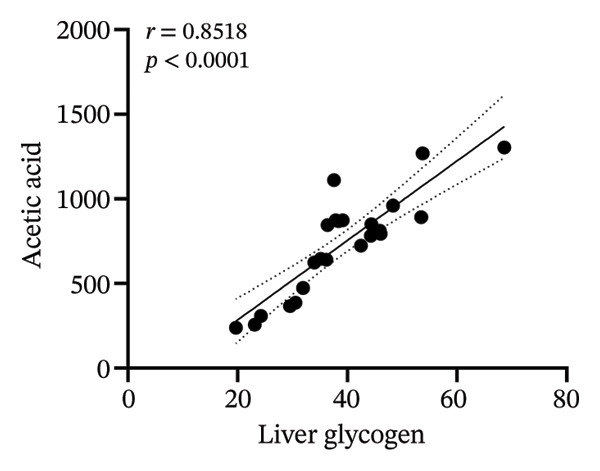
(g)

**Figure:**
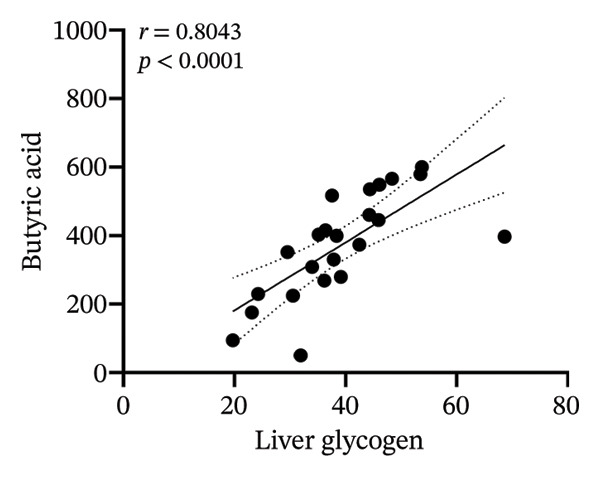
(h)

From the previous 16SrDNA results, the difference in *Bifidobacterium* and *Ammoniphilus* at the genus level was significant between the DM and DM‐NCFMT groups. Therefore, this study conducted a correlation analysis between the differential genera *Bifidobacterium* and *Ammoniphilus* in the DM and DM‐NCFMT groups and the levels of glycemic variability, liver glycogen, acetic acid, and butyric acid, as shown in Figure 11. The correlation coefficients between *Ammoniphilus* and SDBG, LAGE, hepatic glycogen, acetic acid as well as butyric acid were −0.3115, −0.04628, 0.3709, 0.2731, and 0.04979 and *p* values were 0.2082, 0.8553, 0.2576, 0.2728, and 0.8445, respectively). Thus, there was no correlation between *Ammoniphilus* and SDBG, LAGE, hepatic glycogen, acetic acid, and butyric acid. While *Bifidobacterium* had a negative correlation with SDBG (*r* = ‐ 0.7030, *p* = 0.0011), a positive correlation with hepatic glycogen, acetic acid, and butyric acid (*r* = 0.8305, 0.7780, and 0.8196, respectively, *p* = 0.0008, 0.0001, and < 0.0001), and there was no correlation with LAGE (*r* = 0.08925, *p* = 0.7247).

### 3.9. Stepwise Regression Analysis

Despite the correlation analysis showing that differential genera are associated with indicators of blood glucose fluctuations, it still cannot demonstrate that colony transplantation improves blood glucose fluctuations by modulating SCFAs, thereby increasing hepatic glycogen synthesis. The results mentioned above indicate that Bifidobacteria at the genus level shows a significant correlation with the indicators between the DM and DM‐NCFMT groups. Therefore, we used stepwise regression to test if SCFAs and hepatic glycogen mediate Bifidobacteria’s impact on blood glucose fluctuations. Stepwise regression showed that Bifidobacteria’s effect on SDBG does not involve butyric acid and hepatic glycogen, so only meaningful results are presented in this part. Tables 1 and 2 show 95% CI for *Bifidobacterium* abundance ⟶ acetic acid ⟶ liver glycogen ⟶ SDBG pathway excluding 0. It suggests that Bifidobacteria affects SDBG partially via acid and liver glycogen and has a negative correlation with a 23.01% effect share.

**TABLE 1:** Results of the stepwise regression test for the effect of *bifidobacterium* on SDBG via acetic acid and liver glycogen.

	Model 1		Model 2		Model 3		Model 4	
	*β*	*t*	*β*	*t*	*β*	*t*	*β*	*t*
Abundance of *Bifidobacterium*	0.732	3.399	0.604	2.394	−0.845	−4.992	−0.08	−0.619
Acetic acid			0.309	1.224			−0.07	−0.644
Hepatic glycogen							−0.859	−6.433
Adjust *R* ^2^	0.49		0.675		0.685		0.948	
F value	F (1, 10) = 11.556, *p* = 0.007		F (2, 9) = 12.417, *p* = 0.003		F (1, 10) = 24.917, *p* = 0.001		F (3, 8) = 67.653, *p* = 0.000	

**TABLE 2:** Summary of results of the stepwise regression effect sizes of *bifidobacterium* affecting SDBG via acetic acid and liver glycogen.

Route	Effect	Boot SE	Confidence interval (95%)		Proportion of effect (%)
			BootLLCI	BootULCI	
Total effect	−65.624	13.147	−91.391	−39.858	100
Direct effect	−6.223	10.046	−25.913	13.467	9.48
Ind1 *Bifidobacterium* ⟶ acetic acid ⟶ SDBG	−3.997	0.148	−0.731	−0.011	6.09
Ind2 *Bifidobacterium* ⟶ hepatic glycogen ⟶ SDBG	−40.308	0.274	−0.772	0.516	61.42
Ind3 *Bifidobacterium* ⟶ acetic acid ⟶ hepatic glycogen ⟶ SDBG	−15.098	0.249	−1.318	−0.044	23.01

## 4. Discussion

The blood glucose fluctuations in Type 1 diabetes arise from islet *β*‐cell dysfunction, insulin deficiency, reduced glucose utilization by the liver, muscle, and adipose tissues, and decreased hepatic glycogen synthesis. Recent studies have revealed gut flora disorders in T1DM patients, characterized by reduced Bifidobacteria abundance [6], which suggests a role in the regulation of diabetes mellitus and other metabolic diseases. A study showed that healthy individuals’ gut flora transplants can improve glucose tolerance and islet function in diabetics [7, 8]. In a randomized controlled trial in the recent‐onset T1DM, de Groot and colleagues reported that FMT could halt/slow the decline of endogenous insulin production over 12 months, suggesting that microbiota modulation may influence disease trajectory in early‐stage T1DM [9]. Recent studies [10] have shown that after brittle diabetes patients receive intestinal flora transplantation from normal individuals, their blood glucose fluctuations decrease, accompanied by an increase in the *β*‐diversity of intestinal flora, a higher abundance of genus *Prevotella*, and an increase in the butyrate content. Our study found that standard human colony transplantation increased the beta diversity of the gut microbiota, alleviated glucose tolerance, variability, and lipid levels in diabetic rats, confirming its blood glucose‐ and lipid‐lowering effects, which are consistent with prior findings.

A study demonstrated that Bifidobacteria modulate glucolipid metabolism and insulin sensitivity in vivo and in vitro [11]. Jiang demonstrated that *Bifidobacterium longum* fermented milk improves glycolipid metabolism by increasing Bifidobacteria, altering fecal metabolites, and repairing the intestinal barrier [12]. Similar to their results, 16S rDNA sequencing in this study reveals that standard human flora transplantation boosts Bifidobacteria and fecal metabolite acetic acid and ultimately reduces blood glucose fluctuations in T1DM rats.

SCFAs, gut microbial metabolites, sustain gut health, regulate energy balance to improve glucose homeostasis, and enhance insulin secretion, thereby reducing postprandial glucose levels and alleviating diabetes mellitus. For example, Sakakibara et al. found that acetic acid can modulate gluconeogenesis and adipogenesis genes to reduce FBG in diabetic mice [13], while butyric acid promotes energy expenditure and insulin sensitivity and combats insulin resistance [14]. It can also inhibit the process of hepatic gluconeogenesis and improve glucose metabolism. Additionally, Bifidobacteria have been shown to increase acetic acid production [15]. In the present study, the quantities of acetic and butyric acid decreased in diabetic rats, but these quantities increased after normal human intestinal flora transplantation.

Liver glycogen changes are inextricably linked to the progression of diabetes; the liver regulates the dynamic balance of glucose as an insulin‐sensitive organ. It has been reported that *Bifidobacterium longum* improves blood glucose levels in diabetic mice by increasing the hepatic glycogen content [16]. Another study showed that Bifidobacteria increased hepatic glycogen synthesis, decreased the expression of hepatic gluconeogenesis genes, improved the translocation of glucose transporter 4 (GLUT4), and promoted glucose uptake [17]. The study results also showed that liver glycogen in diabetic rats was diminished and reversed by standard human flora transplantation.

Furthermore, the study revealed that intestinal flora transplantation modulated critical enzymes involved in glucose metabolism, thereby enhancing glycogen synthesis in T1DM rats. GK facilitates hepatic glycogen synthesis by modifying glucose to glucose 6‐phosphate [18]. In diabetic patients, the activity of GK is significantly reduced [19]. Jiang reported that *Bifidobacterium longum* can activate glucokinase [12]. As a result, the present study found that GK and hepatic glycogen content were significantly upregulated in the DM‐NCMFT group, suggesting that hepatic glycogen synthesis was reduced in the DM rats. After the intervention of colony transplantation, the diabetic rats exhibited activation of GK, which facilitated blood glucose conversion into hepatic glycogen, thereby improving the liver’s glycogen synthesis and storage capacity and ameliorating hepatic glucose metabolism disorders. Thus, increasing hepatic glycogen synthesis by standard human colony transplantation, which upregulates the activity of GK, may be one of the mechanisms to reduce blood glucose fluctuations in diabetic rats.

To explore whether standard flora transplantation improves abnormal blood glucose fluctuation in T1DM rats via *Bifidobacterium*‐mediated increases in SCFAs, particularly acetic acid, and which upregulate hepatic glycogen synthesis. Correlation and stepwise regression analyses revealed that the reduction of blood glucose fluctuations in diabetic rats is correlated with the increase in the abundance of intestinal Bifidobacteria, acetic acid content, and liver glycogen content.

The present study has several limitations. First, the comparative gut microbiota analysis between healthy humans and normal rats was omitted. Secondly, in the experimental design of our study, we employed a single method to detect various laboratory indicators, such as GK and GP levels in the liver. We did not perform multidimensional detection of their activity, protein expression, or mRNA expression. Thirdly, the FMT donor pool comprised only two patients with fragility diabetes and one healthy donor, which is too small a sample size and may limit the generalizability of the study results and introduce donor‐specific effects. Furthermore, we found that increased Bifidobacteria in the intestine were significantly correlated with decreased blood glucose fluctuations in rats after transplantation of normal human intestinal flora, which was suggested to be partially mediated through hepatic glycogen; however, we did not validate whether the same result could be obtained by infusing a single *Bifidobacterium*. Most importantly, we found that the intestinal flora was closely associated with T1DM blood glucose fluctuations but did not confirm causality or the related signaling pathway mechanisms. Hence, molecular signaling pathway studies are needed to clarify their causality and identify therapeutic targets for managing glucose variability in T1DM.

## 5. Conclusions

Normal human flora transplantation can improve blood glucose fluctuations in T1DM, possibly related to an increase in SCFAs, which are metabolites of gut microbiota, and the synthesis of hepatic glycogen.

## Author Contributions

Min Dou: study design and conduct and writing original draft. Jingwen Xu: data collation and analysis. Xinhua Ye: project administration. Juan Liu: project administration and supervision. Li Shi: project administration, funding acquisition, and validation.

## Funding

This study was supported by grants from the Clinical Research Project of Changzhou Medical Center of Nanjing Medical University (CMCC202310).

## Disclosure

All authors approved it for the final manuscript.

## Ethics Statement

The study was endorsed by the Institutional Animal Care and Use Committee of Nanjing Medical University (IACUC‐2310079).

## Consent

All individuals participating in this study provided informed consent.

## Conflicts of Interest

The authors declare no conflicts of interest.

## Supporting Information

Additional supporting information can be found online in the Supporting Information section.

## Supporting information


**Supporting Information** Table S1 was submitted as supporting information. The patients’ basic information after admission is shown in this table. Figure S1. Effect of FMT on other short‐chain fatty acids in the colonic contents of rats in all groups. (A) Hexanoic acid. (B) Valeric acid. (C) Isobutyric acid. (D) Isovaleric acid. Figure S2. Histogram of TOP10 species composition at the phylum level of rats in all groups. Figure S3. Histogram of LefSe analysis for differential species in all groups (LDA score more significant than 2 for differential flora).

## Data Availability

The original information supporting the assay’s conclusions can be obtained by contacting the author.
