# Supplementary figures and images for: Fecal Microbiota Transplantation From Healthy Donors Reduces Glycemic Variability in Streptozotocin‐Induced Diabetic Rats via Enhanced Hepatic Glycogen Synthesis
Source: Int J Endocrinol. 2026 May 23;2026:8852077. doi: 10.1155/ije/8852077 (PMC13197801; doi:10.1155/ije/8852077)

A

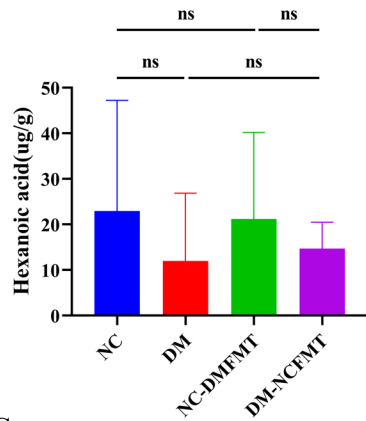

B

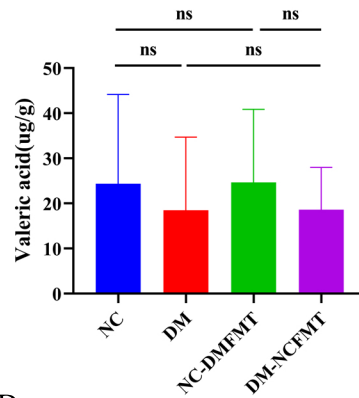

C

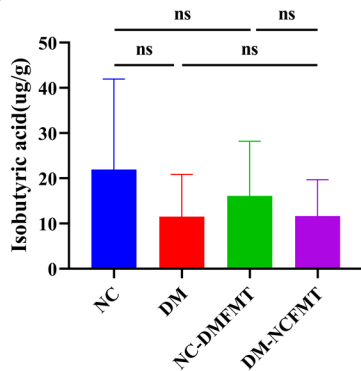

D

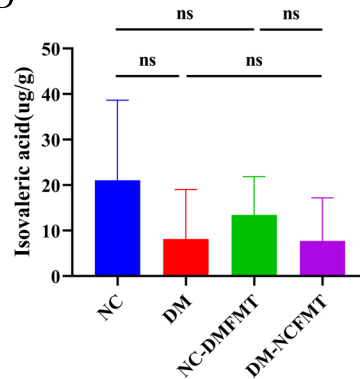

Supplement: Supplementary file 1 — Supporting Information Table S1 was submitted as supporting information. The patients’ basic information after admission is shown in this table. Figure S1. Effect of FMT on other short‐chain fatty acids in the colonic contents of rats in all groups. (A) Hexanoic acid. (B) Valeric acid. (C) Isobutyric acid. (D) Isovaleric acid. Figure S2. Histogram of TOP10 species composition at the phylum level of rats in all groups. Figure S3. Histogram of LefSe analysis for differential species in all groups (LDA score more significant than 2 for differential flora). [file IJE-2026-8852077-s001.zip › Figure S1.pdf]

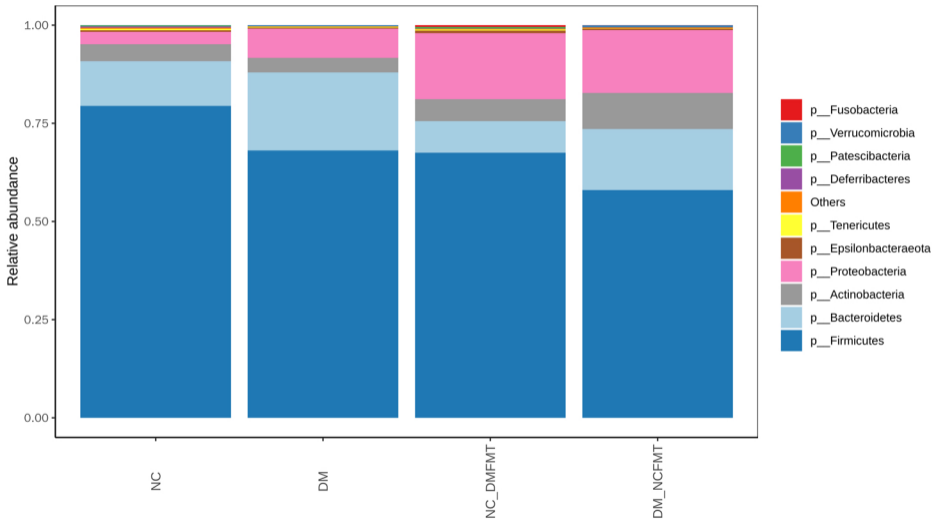

Supplement: Supplementary file 1 — Supporting Information Table S1 was submitted as supporting information. The patients’ basic information after admission is shown in this table. Figure S1. Effect of FMT on other short‐chain fatty acids in the colonic contents of rats in all groups. (A) Hexanoic acid. (B) Valeric acid. (C) Isobutyric acid. (D) Isovaleric acid. Figure S2. Histogram of TOP10 species composition at the phylum level of rats in all groups. Figure S3. Histogram of LefSe analysis for differential species in all groups (LDA score more significant than 2 for differential flora). [file IJE-2026-8852077-s001.zip › Figure S2.pdf]

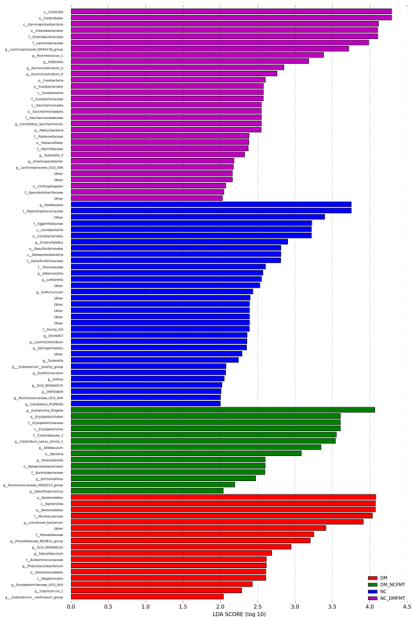

Supplement: Supplementary file 1 — Supporting Information Table S1 was submitted as supporting information. The patients’ basic information after admission is shown in this table. Figure S1. Effect of FMT on other short‐chain fatty acids in the colonic contents of rats in all groups. (A) Hexanoic acid. (B) Valeric acid. (C) Isobutyric acid. (D) Isovaleric acid. Figure S2. Histogram of TOP10 species composition at the phylum level of rats in all groups. Figure S3. Histogram of LefSe analysis for differential species in all groups (LDA score more significant than 2 for differential flora). [file IJE-2026-8852077-s001.zip › Figure S3.pdf]
